# Sacroplasty for Sacral Insufficiency Fractures: Narrative Literature Review on Patient Selection, Technical Approaches, and Outcomes

**DOI:** 10.3390/jcm13041101

**Published:** 2024-02-15

**Authors:** Manjot Singh, Mariah Balmaceno-Criss, Ashley Knebel, Michael Kuharski, Itala Sakr, Mohammad Daher, Christopher L. McDonald, Bassel G. Diebo, John K. Czerwein, Alan H. Daniels

**Affiliations:** 1 Department of Orthopedic Surgery, The Warren Alpert Medical School of Brown University, Providence, RI 02903, USA; 2 Department of Orthopedic Surgery, Hotel Dieu de France, Beirut 166830, Lebanon

**Keywords:** sacral insufficiency fractures, sacroplasty techniques, short-axis, long-axis, coaxial, transiliac, interpedicular, balloon-assisted

## Abstract

Sacral insufficiency fractures commonly affect elderly women with osteoporosis and can cause debilitating lower back pain. First line management is often with conservative measures such as early mobilization, multimodal pain management, and osteoporosis management. If non-operative management fails, sacroplasty is a minimally invasive intervention that may be pursued. Candidates for sacroplasty are patients with persistent pain, inability to tolerate immobilization, or patients with low bone mineral density. Before undergoing sacroplasty, patients’ bone health should be optimized with pharmacotherapy. Anabolic agents prior to or in conjunction with sacroplasty have been shown to improve patient outcomes. Sacroplasty can be safely performed through a number of techniques: short-axis, long-axis, coaxial, transiliac, interpedicular, and balloon-assisted. The procedure has been demonstrated to rapidly and durably reduce pain and improve mobility, with little risk of complications. This article aims to provide a narrative literature review of sacroplasty including, patient selection and optimization, the various technical approaches, and short and long-term outcomes.

## 1. Introduction

Sacral insufficiency fractures are disabling injuries that may occur in the absence of trauma, or result from low-energy injuries such as ground level falls [1]. These fractures can cause severe lower back, buttock, and groin pain and are most commonly identified among older women diagnosed with osteoporosis [2]. Diagnosis can be made using magnetic resonance imaging (MRI), which shows a high-intensity signal indicative of edema on short tau inversion recovery (STIR) sequence. The first line management of these fractures is conservative, consisting of early mobilization, multimodal pain management, activity modification, and osteoporosis management [3]. However, patients that do not respond adequately to nonoperative measures may benefit from sacroplasty or operative fixation [4].

Sacroplasty is a minimally invasive procedure for treating sacral insufficiency fractures that involves placement of polymethylmethacrylate cement into the sacral ala. The cement placement may provide clinically significant pain relief, reducing reliance on pain medication, and improving health-related quality of life [5]. Pain relief can occur in as little as 48 h after surgery, resulting in enhanced functional mobility which, in turn, can reduce the risk of immobility-related complications [6,7]. Sacroplasty is also a relatively safe procedure with low complication profile. The most common complication is cement leakage [8].

A successful sacroplasty requires careful patient selection and optimal surgical technique. There are many technical approaches available, such as transiliac and balloon-assisted, with each having its own benefits and drawbacks. As such, the purpose of this review is to provide an evidence-based assessment of the techniques utilized to perform sacroplasty and present representative clinical cases.

## 2. Pre-Procedure Preparation

### 2.1. Patient Selection

Sacroplasty is generally recommended for patients who have failed conservative management, including bed rest and bracing, have adverse reactions to high-dose analgesics, or are unable tolerate long-term immobilization [9,10,11]. Conservative therapy often results in inadequate pain relief and continued mobility challenges associated with sacral insufficiency fractures. In addition, long-term bedrest can pose significant challenges to frailer patients including deep venous thrombosis, pneumonia, muscular atrophy, fatigue, and others [12,13]. Sacroplasty, on the contrary, allows for better pain management and earlier return to mobility while avoiding the combined impact of prolonged immobilization.

Bone mineral density (BMD) scores should also be considered as part of patient selection. Frey et al., explains that low BMD can lead to chronic nonunion at the fracture site because of impaired ability of osteoporotic bone to heal under strain. Sacroplasty may improve pelvic strength and reduce sacral strain, particularly when pursued in combination with anti-osteoporotic medications [14]. At the same time, it avoids the need for screw and plate fixation in this patient population, which can break through bone and result in loss of fixation [15].

Sacroplasty is contraindicated in patients with uncorrected coagulopathy, local or systemic infection, sacral decubitus ulcers, and allergies to cement [5,16]. In addition, caution should be exercised in patients with gaping fracture lines extending into a sacral foramen or into the dural canal on pre-operative CT since this increases their risk of cement migration into the spinal canal [5].

### 2.2. Classification Systems

Sacral fractures were initially thought to arise from high-energy injuries. As such, they were often classified using the Denis or the AO classification, among others [17]. However, with advanced imaging now showing a higher prevalence of osteoporotic- and stress-related sacral fractures, management based solely on these classification systems is no longer widely agreed upon. This is because these systems emphasize high-energy traumatic mechanisms, often with associated neurologic or pelvic ring injuries, that may warrant more aggressive interventions such as sacroiliac screws or spinopelvic fixation, than sacral insufficiency fractures [18]. Appropriate decision-making between surgical or non-surgical management requires an understanding that sacral insufficiency fractures frequently present in older patients with low bone mineral density following low energy injuries and, as such, create progressive instability from the accumulation of additional fractures [19]. Classification schemes unique to this population have thus been generated.

**Figure 1:**
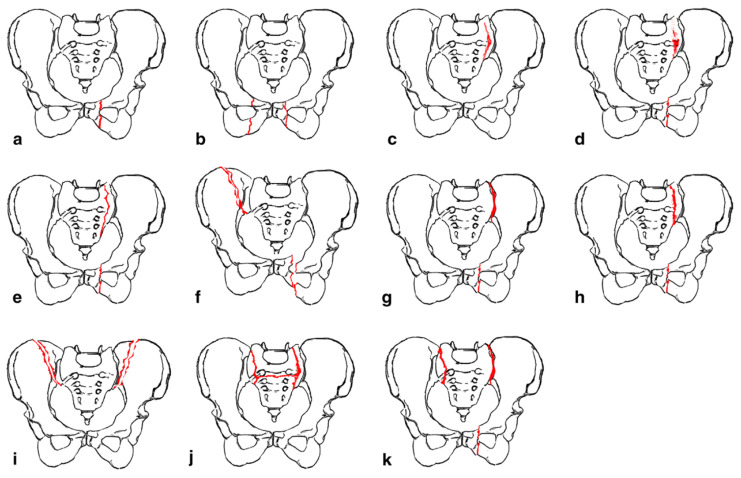
“ Classification of fragility fractures of the pelvis. (**a**) FFP Type Ia: unilateral anterior pelvic ring disruption. (**b**) FFP Type Ib: bilateral anterior pelvic ring disruption. (**c**) FFP Type IIa: dorsal non-displaced posterior injury only. (**d**) FFP Type IIb: sacral crush with anterior disruption. (**e**) FFP Type IIc: non-displaced sacral, sacroiliac or iliac fracture with anterior disruption. (**f**) FFP Type IIIa: displaced unilateral ilium fracture and anterior disruption. (**g**) FFP Type IIIb: displaced unilateral sacroiliac disruption and anterior disruption. (**h**) FFP Type IIIc: displaced unilateral sacral fracture together with anterior disruption. (**i**) FFP Type IVa: bilateral iliac fractures or bilateral sacroiliac disruptions together with anterior disruption. (**j**) FFP Type IVb: spinopelvic dissociation with anterior disruption. (**k**) FFP Type IVc: combination of different posterior instabilities together with anterior disruption” by Rommens et al. (Accessed 12 December 2023 at https://doi.org/10.1007/s00776-014-0653-9). Licensed under CC BY-NC-ND 4.0 © 2014 The Japanese Orthopaedic Association. To view a copy of this license, visit https://creativecommons.org/licenses/by-nc-nd/4.0/ (Red color indicates fracture lines).

Rommens and Hofmann proposed the fragility fractures of the pelvis (FFP) classification to guide management of fractures occurring anywhere in the pelvis [19]. The FFP classification has four main types with fracture displacement being a key distinguishing factor between the types (Figure 1). FFP Type I often deals with fractures of the anterior pelvic ring while FFP Types II-IV encompass posterior fractures with and without concomitant anterior fractures. FFP Type II encompasses nondisplaced fractures while Types III and IV encompass unilateral and bilateral displaced posterior fractures, respectively. This system recommends conservative treatment for FFP Type I and II fractures [20,21]. However, close monitoring is recommended for Type II as supplemental percutaneous fixation with screw placement or sacroplasty may be required. FFP Types III and IV necessitate more aggressive operative management and may be augmented with sacroplasty.

**Figure 2:**
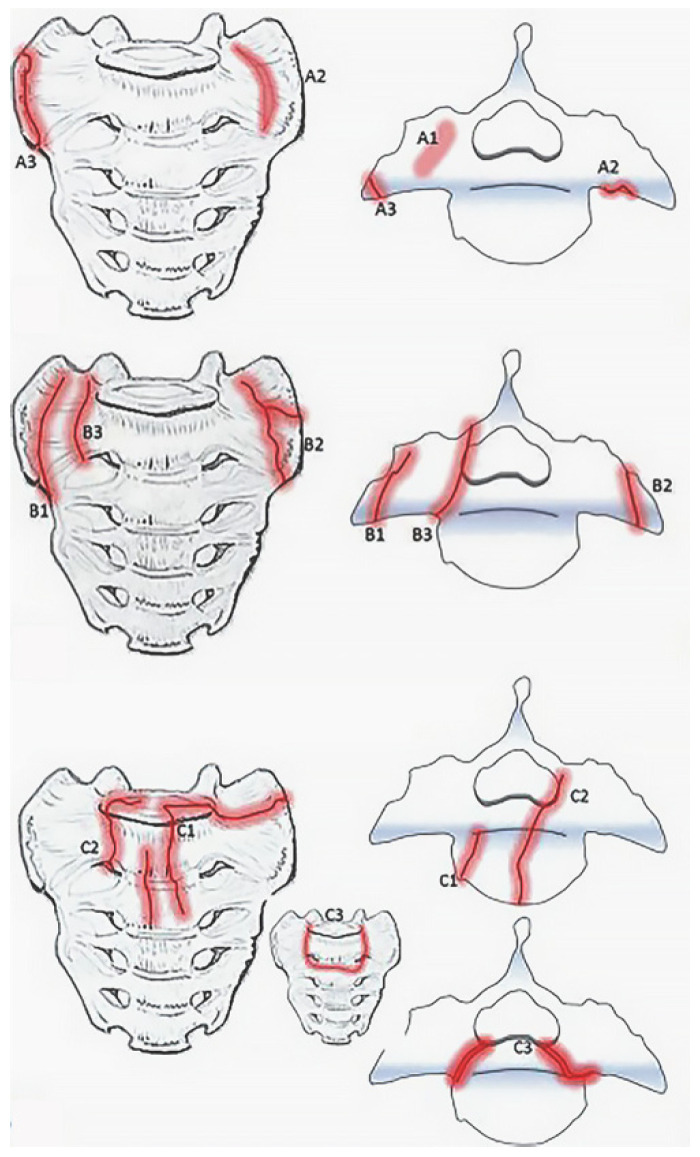
Bakker classification for sacral insufficiency fractures adapted from “Type A-fractures of the sacral ala: A1 with bone bruise (MRI) without a visible fracture line in the CT-scan; A2 deformation of the anterior cortical bone without a cortical disruption; and A3 anterolateral rim fracture of the ala with up to 1 cm distance in the direction of the medial sacroiliac joint.” (**top**), “ Type B fractures of the sacral ala: B1 fracture parallel to the sacroiliac joint; B2 fracture involving the sacroiliac joint; and B3 fracture with an involvement of the neural foramina or the spinal canal.” (**middle**), and “ Type C- or corpus-fractures: C1 fracture moves from anterior cortex dorsally or into the sacroiliac joint; C2 fracture with an unilateral involvement of the neural foramina or the spinal canal; and C3 is unstable and represents bilaterally sagittal fractures combined with a transverse lesion.” (**bottom**) by Bakker et al. (https://doi.org/10.3340/jkns.2017.0188). Original figures licensed under CC BY-NC-ND 4.0© 2018 The Korean Neurosurgical Society. To view a copy of this license, visit https://creativecommons.org/licenses/by-nc-nd/4.0/ (Red indicates fracture lines).

Bakker et al., recently developed the Bakker classification which classifies sacral insufficiency fractures first by region, ala or corpus, and then by associated characteristics such as involvement of the sacroiliac joint or neural foramina (Figure 2) [22,23]. Type A are localized to the sacral ala while type B sacral ala fractures and type C corpus fractures may extend to the sacroiliac joint, neuroforamina, or spinal canal [22]. In a subsequent small validation study, conservative management was sufficient for type A fractures and one third of the type B fractures [23]. The rest of the type B fractures required percutaneous screw fixation or sacroplasty. However, since the study did not classify by subtype, it is not clear whether failure of conservative management in type B fractures could be attributed to involvement of the sacroiliac joint or the neuroforamina. In addition, the study was underpowered to make a definitive treatment recommendation for type C fractures.

It is important to note that despite the development of the Rommens and Hofmann and the Bakker classifications, these have yet to be externally validated on a large subset of patients with prospective studies. Thus, a suitable evidence-based treatment algorithm for sacral insufficiency fractures remains elusive.

### 2.3. Pre-Procedural Optimization

Pain management is an essential part of both conservative and operative management of sacral insufficiency fractures. Multimodal pain management should be pursued to avoid over utilization of potent opioids in this patient population [24]. Additionally, medical optimization of bone health with anti-osteoporotic medications prior to operative intervention should also be considered. For instance, bisphosphonates can be administered to osteoporotic patients awaiting sacroplasty. Bisphosphonates increase early bone growth and improve bone mineral density, which can reduce the risk of vertebral fractures post-operatively [25]. Furthermore, many patients who suffer sacral insufficiency fractures are also vitamin D deficient and may benefit from pre-operative vitamin D supplementation, which has been shown to help reduce the risk of pseudoarthrosis [26]. Finally, hormonal supplementation can improve post-operative healing, reduce bone pain, and analgesic reliance. Calcitonin has been shown to reduce osteoporotic bone pain and is typically used acutely for this purpose [27]. While calcitonin is anti-osteoporotic, its effects are minimal, so it is not typically used as first-line treatment for long-term medical management [28]. Instead, teriparatide, an osteoanabolic agent that is a synthetic form of human parathyroid hormone, has been utilized in postmenopausal women with osteoporosis [29]. Teriparatide promotes new bone formation and remodeling through activation of osteoblasts [30]. Use of teriparatide has been shown to reduce pain, facilitate early mobilization, and promote direct healing in patients undergoing sacroplasty, without increasing rates of primary bone malignancies which has previously been a concern with this medication [31,32]. Denosumab and Romosozumab, other osteoanabolic agents, have shown an even greater BMD improvement at the lumbar spine and hip through blockade of the inhibitory effects of sclerostin, which results in an increase in bone formation and decrease in bone resorption [33,34]. Multiple studies have demonstrated superiority of Romosozumab compared to Denosumab for improving BMD at twelve months [35]. However, compared to Denosumab, treatment with Romosozumab is limited to 12 months and cessation is associated with rapid loss of its effects on BMD [36,37]. Thus, it is recommended that patients begin another antiresorptive therapy after Romosozumab discontinuation. Despite this limitation, treatment with Romosozumab prior to other antiresorptive medications has been shown to result in greater gains in BMD, making this treatment sequence favorable [38]. The use of one or a combination of these medication classes prior to and in conjunction with sacroplasty may help improve patient outcomes.

### 2.4. Patient Positioning

In preparation for sacroplasty, patients are placed in the prone position, with a pillow under the pelvis to elevate the sacrum. Bony landmarks, including the L5-S1 disc, S1 and S2 neuroforamina, and sacroiliac joint, are marked using conventional fluoroscopy. Visualization of these landmarks on fluoroscopy is demonstrated in patient case one. Computed tomography and/or navigation can then be utilized to better visualize the sacral anatomy.

### 2.5. Material Considerations

Materials required for sacroplasty, at bare minimum, include spinal needles, polymethylmethacrylate (PMMA) bone cement, and cement application tools. Use of balloon-assistance, discussed below, may also be considered. The biomechanics of cement injection and the associated risk of cement leakage depend on cement viscosity. A small and safe amount of high-viscosity cement, achieved by increasing the time elapsed since mixing or the powder-to-liquid ratio, injected using a small diameter needle yields the lowest risk of cement leakage [8]. Cumulative procedural costs, not accounting for operating room time and other hospital costs, are approximately $5521–$5784 [39,40].

## 3. Procedure Techniques

### 3.1. Bilateral Short Axis

**Figure 3:**
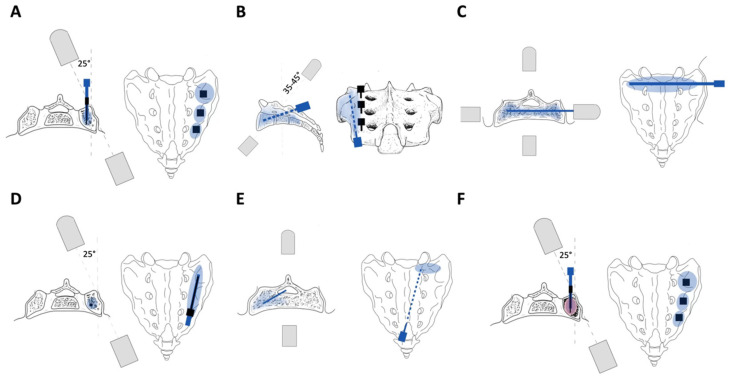
Sacroplasty surgical techniques: (**A**) bilateral short axis, (**B**) bilateral long axis, (**C**) coaxial vision, (**D**) transiliac, (**E**) interpedicular, (**F**) balloon-assisted. (Blue color denotes the site of cement deposition. Red color denotes the space created by balloon assistance).

In this approach, initially described by Garant et al., the fluoroscope is first set to anteroposterior (AP) view with a 25–30-degree contralateral oblique tilt to visualize the medial and lateral aspects of the sacroiliac joint [41]. A 22-gauge spinal needle is placed between the S1 portion of the sacroiliac joint and the lateral margin of the S1 neural foramen and directed towards the center of the S1 body. The same technique is then repeated for each sacral level. Finally, 11-guage needles are advanced into each sacral body and slowly retracted as cement is placed (Figure 3A). The short-axis approach offers localized placement of cement along the fracture site. However, appropriate placement of the needle tip into the intramedullary space of the sacral ala without breaching the anterior cortex can be difficult to achieve. Furthermore, the volume of cement that can be injected is often limited, with frequent extravasation of the cement early in the injection [42].

### 3.2. Bilateral Long Axis

In this approach, initially described by Smith et al., the fluoroscope is first set to AP view with a 25–30-degree contralateral oblique tilt to visualize the medial and lateral aspects of the sacroiliac joint [42]. A 22-gauge spinal needle is placed between the inferior margin of the sacroiliac joint and the lateral margin of the S3 neural foramen and directed towards the center of the superior margin of the sacroiliac joint and the lateral margin of the S1 neural foramen. An 11-guage needle is finally advanced into the S1 sacral body and slowly retracted as cement is placed from S1 to S3 (Figure 3B). The long-axis approach offers enhanced distribution of the cement along the vertical length of a sacral fracture and reduced risk of cement extravasation produced by inadvertent perforation of the anterior cortex during the short-axis approach [43]. However, breaching of the anterior cortex is still possible and penetration of the superior margin of the ala could also occur, either of which could lead to cement extrusion into the adjacent soft tissues or the sacral neuroforamina.

### 3.3. Coaxial Vision

In this approach, initially described by Silva-Ortiz et al., the fluoroscope is first set to AP view with a 15-degree cephalad tilt to identify the lateral limit of the S1 foramen [44]. The fluoroscope is then adjusted to a 35–45-degree caudad tilt to align the anterior and posterior aspect of the sacrum, thereby giving the coaxial view of the sacral bone. A 22-gauge spinal needle is placed between the sacroiliac joint line and the sacral foramina. An 11-gauge needle is finally advanced into the S1 sacral body and slowly retracted as cement is placed from the S1 to the S3 vertebral bodies (Figure 3C). The coaxial vision has the benefit of being less technically challenging and significantly decreases the risk of cement extravasation through accurate identification of the anterior sacral cortical bone. However, comparative studies assessing the utility and effectiveness of this approach have not been conducted and the risks associated with the previously described approaches remain.

### 3.4. Transiliac

In this approach, initially described by Nicholson et al., the fluoroscope is first set to AP and lateral views, and adjusted to visualize the S1 neural foramina [45]. An 11-gauge needle is then advanced at the level of S1 transversely across the ilium, sacroiliac joint, and sacrum until it reaches the contralateral sacral ala. The needle is slowly retracted as cement is placed within the intramedullary cavity between the anterior and posterior cortices of the bone (Figure 3D). The transiliac approach allows for cement deposition in the intramedullary space of both sacral ala with a single incision and buttressing of the fracture along either side of the fracture line. At the same time, it minimizes penetration of the anterior cortex of the sacrum. However, the complex anatomy of the sacral bone, with nearby pelvic organs and neurovascular structures, often necessitates a good understanding of complex sacral morphology and anatomy on plain radiographs or use of advanced imaging to prevent needle malpositioning.

### 3.5. Interpedicular

In this approach, initially described by Firat et al., the fluoroscope is first set to an AP view and rotated until the sacral neuroforamina can be visualized [46]. A 13-guage needle is placed over the sacral hiatus at the level of the third or fourth sacral vertebral body, and directed parallel to the long axis of the sacrum until it passes the posterior wall of the sacral spinal canal and reaches the anterior border of the sacral canal at the desired sacral vertebral level. The needle is slowly retracted as cement is placed from the S1 to the S3 or the S3 to S5 vertebral bodies (Figure 3E). The interpedicular approach allows for access to the lower sacral vertebrae and significantly reduces the risk of cement extravasation into the neural foramina. However, there is a risk of nerve root injury and epidural infection while traversing the spinal canal.

### 3.6. Balloon-Assisted

In this approach, initially described by Andresen et al., the sacrum is first accessed through one of the approaches described above [47]. A 15-mm balloon is then inserted through the trocar and inflated to generate an intramedullary void. The balloon is subsequently deflated and cement is finally deposited to fill the cavity similar to a kyphoplasty (Figure 3F). Balloon assistance allows for compaction of the fractured bone and offers greater cement deposition at the fracture site, reportedly leading to higher pelvic stabilization and pain relief [48]. Studies have further shown lower risk of cement extravasation since balloon assistance allows for controlled cement introduction. However, it does increase operation time and costs, and may still result in occasional cement leakages.

## 4. Post-Operative Outcomes

### 4.1. Short-Term Outcomes

#### 4.1.1. Pain Management

Sacral insufficiency fractures may cause significant lower back, buttock, and groin pain. Pain relief following sacroplasty is often rapid in onset and sustained even months after the procedure [49,50]. In a cohort of 52 patients, Frey et al., showed improvement in mean visual analog scale (VAS) pain score from 8.1 at baseline to 3.4 within thirty minutes of the procedure, and finally 0.8 at one year follow up [49]. In another cohort of 102 patients, Beall et al., found improvement in the mean numerical rating scale (NRS) score, decreasing from 7.8 pre-operative to 0.9 six months after the procedure [50]. Additionally, reliance on narcotic pain medications also decreases significantly post-operatively. Such success in pain control is likely attributed to reduced fracture-associated mechanical stress and micromotion following sacroplasty [51].

#### 4.1.2. Functional Mobility

Sacroplasty has been shown to improve functional mobility and ability to perform activities of daily living within weeks of the procedure [50,52,53]. In a cohort of 102 patients, Beall et al., showed improvement in the mean Roland Morris Disability Questionnaire (RMDQ) score from 17.7 to 5.2 at six months following the procedure [50]. In a smaller study on 16 patients, Choi et al., noted a reduction in the mean Oswestry Disability index (ODI) score from 59.0 pre-operatively to 14.8 at three month follow up [52]. Likewise, Talmadge et al., showed clinical mobility scale (CMS) scores improved from 8.40 at baseline to 17.53 forty-eight weeks after the procedure in a study on 18 patients [53]. This is in contrast to standard conservative management which can take up to 12 months for patients to heal and achieve optimal functional status. The mechanism of recovery is likely similar to what has been described previously, with sacroplasty offering decreased micromotion and increased pelvic stabilization for pain-free motion [51].

### 4.2. Long-Term Outcomes

Long-term studies suggest that the positive effects of sacroplasty, such as sacral pain relief and enhanced mobility, persist even ten years after the procedure [54]. Patient satisfaction at one year follow-up also remains high, with patients stating that they would repeat the procedure in the event of similar pain episodes [55].

### 4.3. Complications

Sacroplasty is generally safe and efficacious, with a major complication rate as low as 0.3% [9]. The most common complications, as noted by Mahmood et al., include clinically insignificant cement extravasation, persistent lower back or new-onset radicular leg pain, compression fractures, and reoperations [12]. Cement leakage is reported in 2.2–3.3% of cases and occurs mainly through the fracture gap, resulting in compression of the L5 nerve root and radicular pain symptoms [8,9]. Careful needle placement, use of intraoperative imaging, and balloon assistance can often reduce the rate of cement leakage, although this cannot be completely prevented [48]. Subsequent neurologic symptoms are reported in 0.4–0.6% of cases and require anti-inflammatory medications, nerve root block, or surgical decompression for management [9,56]. Other operative complications, such as needle trauma, intravascular injection, hematoma or bleeding, and infection, have also been reported but are rare and not unique to sacroplasty alone [14]. These complications should be weighed against the potential complications of conservative management, including deep vein thromboses, pulmonary emboli, and reduced muscle strength or breakdown following prolonged inactivity to identify the appropriate treatment plan for the patient [14].

## 5. Illustrative Cases

### 5.1. Case Selection

Patient cases were included in this article if they could provide informed consent, met the surgical indications for sacroplasty, underwent sacroplasty through one of the technical approaches described above, and had pre- and post-operative imaging available.

### 5.2. Case One

**Figure 4:**
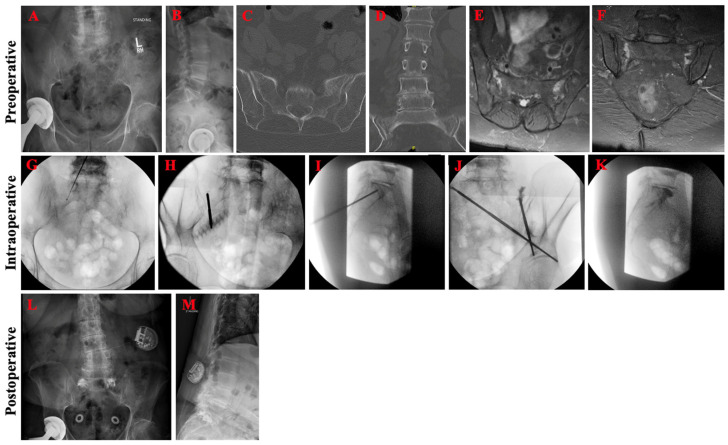
Pre-operative, intraoperative, and post-operative imaging for patient case 2. Pre-operative anteroposterior (**A**) and lateral (**B**) lumbosacral spine radiographs demonstrated spondylosis of L3 over L4. Axial (**C**) and coronal (**D**) CT confirmed L3–4 dynamic spondylolisthesis and bilateral sacral alar insufficiency fractures. Axial I and sagittal (**E**,**F**) MRI STIR sequence of a related patient case showing edema at the fracture site. Intraoperative imaging during bilateral long-view sacroplasty demonstrated (**G**) successful ball tip probe cannulation of the right sacral ala without anterior penetration of the sacrum on anteroposterior inlet view, (**H**) Jamshidi cannulation down the right SI joint with no joint penetration on 25° right oblique view, (**I**) Jamshidi directed towards the anterior vertebral body with appropriate trajectory on lateral view, (**J**) cement filling the left sacral ala without joint or foraminal penetration on left oblique view, and (**K**) no final anterior or superior extravasation of cement on lateral view. Post-operative anteroposterior (**L**) and lateral (**M**) lumbosacral spine radiographs re-demonstrated spondylosis of L3 over L4 and good distribution of the cement without extravasation.

An 84-year-old female with a history of osteoporosis and chronic back pain presented with ten days of progressively worsening left-sided lower back and radicular leg pain, as well as difficulty with ambulation. She denied any recent history of trauma. She had previously trialed and failed conservative management with physical therapy, pain medications, and epidural steroid injections. Four-view lumbosacral spine radiographs demonstrated dynamic spondylolitic changes of the L3–4 vertebrae (Figure 4A,B). Lumbosacral CT confirmed L3–4 dynamic spondylolisthesis and bilateral Bakker B1 sacral alar insufficiency fractures (Figure 4C,D). Lumbar and pelvic MRI redemonstrated CT findings and confirmed severe stenosis at the L3–4 level, consistent with her radicular leg symptoms. In addition to L3–L4 posterior lumbar decompression and fusion, bilateral sacroplasty through the bilateral long-axis approach was pursued without cement extravasation or other complications (Figure 4G–K). Post-operatively, pelvic radiographs showed adequate cement deposition at the fracture site (Figure 4L,M). Patient had marked improvement in pain (ODI from 50 to 34, VAS from 9 to 4) and was able to mobilize independently on post-operative day one.

### 5.3. Case Two

**Figure 5:**
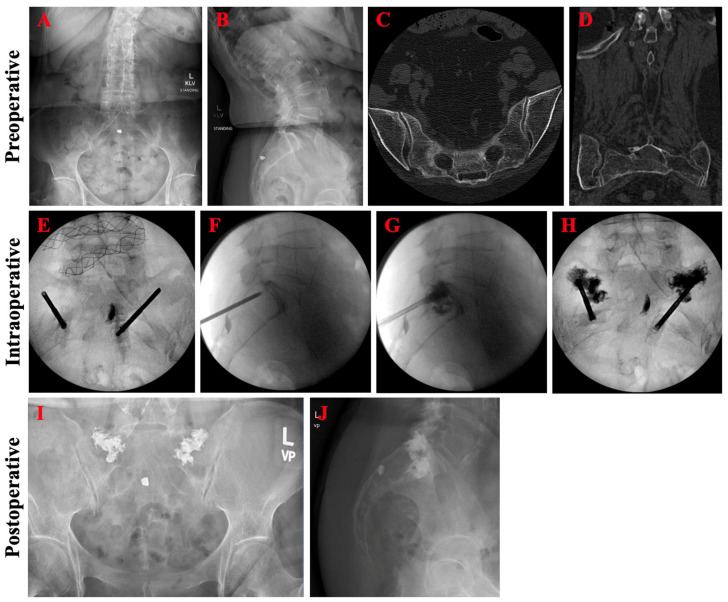
Pre-operative, intraoperative, and post-operative imaging for patient case 1. Pre-operative anteroposterior (**A**) and lateral (**B**) lumbosacral spine radiographs demonstrated degenerative spondylolitic changes of the lumbar vertebrae. Axial (**C**) and coronal (**D**) CT confirmed bilateral sacral fractures centered around S3. Intraoperative (**E**–**H**) imaging during bilateral short-view sacroplasty revealed adequate cement deposition at the fracture site. Post-operative anteroposterior (**I**) and lateral (**J**) lumbosacral spine radiographs showed good distribution of the cement without extravasation.

A 73-year-old female with a history of lumbar spinal stenosis presented with three weeks of lower back and bilateral hip/groin pain after falling off her bed and landing on her buttocks. Rest, ice, and over-the-counter pain medications had been ineffective thus far and she had become reliant on a cane for ambulation. Four-view lumbosacral spine radiographs demonstrated degenerative spondylolitic changes of the lumbar vertebrae (Figure 5A,B). Lumbosacral CT revealed bilateral Bakker B3 sacral fractures centered around S3 (Figure 5C,D). Bilateral sacroplasty through the bilateral short-axis approach was pursued without cement extravasation or other complications (Figure 5E–H). Post-operatively, pelvic radiographs showed adequate cement deposition at the fracture site (Figure 5I,J). Patient had marked improvement in pain (VAS from 8 to 5) and early return to ambulation independent of assistive devices.

## 6. Conclusions

Sacral insufficiency fractures are stress fractures of the sacral ala that can cause severely disabling lower back pain, especially in the elderly diagnosed with osteoporosis. Sacroplasty is a minimally invasive procedure used for the treatment of these fractures that involves placement of polymethylmethacrylate cement around the fracture site. There are many surgical approaches available, including short-axis, long-axis, coaxial, transiliac, and interpedicular. Regardless of the approach, sacroplasty offers good short-term and long-term clinical outcomes with minimal complications if performed properly.

## Data Availability

Not applicable.
